# EFFECTIVENESS OF ORAL MEDICATIONS IN THE PERIOPERATIVE PERIOD OF ROTATOR CUFF INJURIES - SYSTEMATIC REVIEW

**DOI:** 10.1590/1413-785220253302e286067

**Published:** 2025-06-02

**Authors:** Edivando de Moura Barros, Elzir Finizola Costa, Fabio Teruo Matsunaga, Milla Pompilio da Silva, Eiffel Tsuyoshi Dobashi, Marcel Jun Sugawara Tamaoki

**Affiliations:** 1 Universidade Federal de Sao Paulo, Escola Paulista de Medicina, Departamento de Ortopedia, Grupo de Ombro e Cotovelo, Sao Paulo, SP, Brazil.; 2 Universidade Federal de Sao Paulo, Escola Paulista de Medicina, Sao Paulo, SP, Brazil.; 3 Universidade Federal de Sao Paulo, Escola Paulista de Medicina, Departamento de Ortopedia, Sao Paulo, SP, Brazil.

**Keywords:** Perioperative Period, Postoperative Care, Pain, Rotator Cuff Injuries, Drug Therapy, Período Perioperatório, Cuidados Pós-Operatórios, Dor, Lesões do Manguito Rotador, Terapia Medicamentosa

## Abstract

Postoperative pain management remains a significant challenge in patient care, being particularly crucial in the context of arthroscopic or open rotator cuff repairs.

**Objective:**

To compare the effectiveness of different medications in controlling pain after this surgical intervention.

**Material and methods:**

A systematic review of the literature was carried out, conducting a data search in the Pubmed/Medline, Science Direct and Embase databases. Initially, 1,223 articles were identified where, after detailed analysis, eight studies were selected to compose this research. The total sample comprised 703 patients.

**Results:**

We observed a predominance of male participants in most studies, and the age range of those involved varied, on average, from 50.0 to 59.9 years old. The studies included cover a variety of pain management strategies after arthroscopic rotator cuff repair, highlighting the diversity of approaches in the literature, including analgesics, anti-inflammatories, anticonvulsants and opioids.

**Conclusion:**

In general, we showed that the interventions used were well tolerated. Some studies have demonstrated mild adverse effects, such as nausea, vomiting, dizziness, headache and other transient symptoms. This systematic review highlights that there is no well-established protocol for managing postoperative pain in arthroscopic rotator cuff repair, comparing the effectiveness of different medications. The diversity of approaches observed in the literature highlights the need for personalized strategies. Despite the good tolerance and positive results of the interventions, the choice of medication must consider the individuality of the patient and specific characteristics of the procedure. ***Level of Evidence II; Systematic Review.***

## RESUMO

O gerenciamento da dor pós-operatória permanece como um desafio significativo na prestação de cuidados ao paciente, sendo particularmente crucial no contexto do reparo artroscópico ou aberto do manguito rotador.

**Objetivo:**

Comparar a efetividade de diversas medicações no controle da dor após essa intervenção cirúrgica.

**Material e métodos:**

Realizou-se uma revisão sistemática da literatura, conduzindo a busca de dados nas bases Pubmed/Medline, Science Direct e Embase. Foram identificados 1.223 artigos, oito estudos foram incluídos. A amostra total compreendeu 703 pacientes.

**Resultados:**

Os participantes foram do sexo masculino na maioria dos estudos, e a faixa etária dos envolvidos variou, em média, de 50,0 a 59,9 anos. Os estudos incluídos abarcam uma variedade de estratégias de manejo da dor após o reparo artroscópico do manguito rotador, destacando a diversidade de abordagens existentes na literatura, incluindo medicamentos analgésicos, anti-inflamatórios, anticonvulsivantes e opioides.

**Conclusão:**

De maneira geral, evidenciamos que as intervenções empregadas foram bem toleradas. Alguns estudos demonstraram efeitos adversos leves, como náuseas, vômitos, tontura, cefaleia e outros sintomas transitórios. O uso pós-operatório de ibuprofeno reduz a necessidade de opioides e diminui os níveis de dor do paciente na primeira semana pós-operatória e sua utilização não aumentou o risco de rerrotura. ***Nivel de Evidência II; Revisão Sistemática.***

## INTRODUCTION

Rotator cuff injury is the leading cause of upper limb dysfunction related to the shoulder.^1^ The symptoms of these injuries are characterized by pain, predominantly located in the front or lateral part of the shoulder, which may intensify with specific movements.^2^

The management of rotator cuff injuries is the subject of extensive discussion, encompassing both non-surgical and surgical approaches. Among the non-surgical options, we can mention modifications to daily activities, the use of medications for symptom relief, and participation in physical therapy programs.^3^

The number of patients undergoing rotator cuff repair (RCR) has increased, and postoperative pain control is considered a challenge in these cases. Some studies show an increase in this type of procedure ranging from 163% to 268%.^4^

A national study utilized the DataSUS table, which is based on population data from the Brazilian Institute of Geography and Statistics (IBGE). The rates presented per group of 100,000 inhabitants, from 2003 to 2017, regarding rotator cuff tear repair, recorded 50,207 surgeries, including decompression procedures. The rates were calculated using the total number of rotator cuff repairs as the numerator and the total population of the evaluated locality as the denominator. The rate of surgical interventions increased from 0.83 to 2.81, resulting in a 238% rise. In 2015, the South Region had the highest rate, at 6.32, followed by the Southeast, at 3.62, while the North had the lowest rate, at 0.13. The trend of this growing expansion is observed in the Southeast, South, and Midwest regions, while the index is stable in the North and Northeast regions.^5^

Despite being a minimally invasive surgical procedure, it is associated with significant pain observed in the immediate postoperative period, especially in the first 2-3 days after surgery. However, effective pain control at this time can help reduce hospital stays and improve patient satisfaction and functional recovery.^6^

There is no systematic review of postoperative pain control for rotator cuff repair managed solely with oral medications. In this perspective, the present study aims to investigate the influence of different medications on postoperative pain control and evaluate which medication would have the greatest effectiveness in patients undergoing RCR.

The importance of this work lies in the fact that, despite numerous therapeutic approaches having been described for pain control, this topic remains a significant issue for both patients and orthopedic surgeons.

The objective is to compare the effectiveness of different medications administered orally for pain control in patients undergoing RCR and the specific objectives are: To conduct a comprehensive literature analysis to evaluate the effectiveness of analgesics, anti-inflammatories, antidepressants, anticonvulsants, and opioids; to investigate and consolidate existing data addressing the effectiveness and adverse effects of oral medications used, and to perform a critical and comprehensive synthesis of existing evidence, contributing to a deeper understanding of this topic.

## METHODOLOGY

This is a systematic literature review registered in the International Prospective Register of Systematic Reviews (PROSPERO) database. The review was conducted in accordance with the Preferred Reporting Items for Systematic Reviews and Meta-Analyses (PRISMA) guidelines.

The research was guided by the following question: "What is the most effective oral medication for postoperative pain control in patients undergoing rotator cuff repair?" This question was formulated according to the PICO strategy. (Table 1)

**Table 1:** The PICO strategy was adopted to define the guiding question of the research.

P – Population	Patients in the perioperative period of rotator cuff repair
I – Intervention	Oral medication
C – Comparison	Compare the effectiveness of medications
O – Outcome	Pain, function, adverse effects

Source: Author (2024).

The following databases were used: PubMed, ScienceDirect, and Embase. The search strategy included terms related to rotator cuff repair, postoperative pain medications, and randomized clinical trials. The research strategy is presented in Table 2.

**Table 2:** Search strategy for the review articles.

Pubmed Central	("rotator cuff"[MeSH Terms] OR ("rotator"[All Fields] AND "cuff"[All Fields]) OR "rotator cuff"[All Fields]) AND ("wound healing"[MeSH Terms] OR ("wound"[All Fields] AND "healing"[All Fields]) OR "wound healing"[All Fields] OR "repair"[All Fields]) AND ("pain, postoperative"[MeSH Terms] OR ("pain"[All Fields] AND "postoperative"[All Fields]) OR "postoperative pain"[All Fields] OR ("postoperative"[All Fields] AND "pain"[All Fields])) AND ("pharmaceutical preparations"[MeSH Terms] OR ("pharmaceutical"[All Fields] AND "preparations"[All Fields]) OR "pharmaceutical preparations"[All Fields] OR "medications"[All Fields]) AND ("pain management"[MeSH Terms] OR ("pain"[All Fields] AND "management"[All Fields]) OR "pain management"[All Fields] OR ("pain"[All Fields] AND "control"[All Fields]) OR "pain control"[All Fields]) AND ("randomized controlled trial"[All Fields] OR "randomized controlled trials as topic"[MeSH Terms] OR "randomized clinical trials"[All Fields] OR "randomized clinical trials"[All Fields])
Science Direct	(‘Rotator Cuff Repair’) AND (‘postoperative pain medications’ OR (postoperative AND medications)) AND (‘pain control’/exp OR ‘pain control’) AND (‘randomized controlled trial’ OR ‘randomized controlled trials as topic’)
Embase	(‘rotator cuff repair’/exp OR ‘rotator cuff repair’ OR ((‘rotator’/exp OR rotator) AND (‘cuff’/exp OR cuff) AND (‘repair’/exp OR repair))) AND (‘postoperative pain medications’ OR (postoperative AND (‘pain’/exp OR pain) AND medications)) AND (‘pain control’/exp OR ‘pain control’ OR ((‘pain’/exp OR pain) AND (‘control’/exp OR control))) AND (‘randomized clinical trials’ OR (randomized AND (‘clinical’/exp OR clinical) AND trials))

Source: Pubmed Central, Science Direct, Embase (2024).

A comprehensive review of the literature was conducted to identify and include randomized clinical trials investigating oral pharmacological interventions for postoperative control in individuals undergoing rotator cuff repair. Covering all age groups and both sexes, with no ethnic or age restrictions, the review included various medications such as analgesics, non-steroidal anti-inflammatory drugs (NSAIDs), corticosteroids, opioids, antidepressants, anticonvulsants, and supplements. Studies whose interventions did not exclusively involve the use of oral medications in the postoperative period were excluded. The primary outcome is to assess pain intensity (using a VAS or another tool), and the secondary outcomes include assessing adverse effects, function, quality of life, use of rescue analgesics, and tendon rerupture.

Initially, two independent reviewers analyzed the titles and abstracts of the studies identified in the search. Those that met the inclusion criteria were selected for full review. Relevant data were extracted from the selected studies, including characteristics, population, interventions, and outcomes related to postoperative pain. Any disagreements between the reviewers were resolved by consensus or, when necessary, by consulting a third reviewer. This approach aimed to ensure consistency and reliability in the selection and analysis of studies, thereby strengthening the validity and accuracy of the results obtained in the systematic review, a process that has already been completed.

The methodological quality of the included randomized clinical trials was assessed through an evaluation of the risk of bias for each study using the ROB-2 tool, following the criteria described in Chapter 8 of the Cochrane Handbook for Systematic Reviews of Interventions.^7^ Any discrepancies were resolved through discussion or by involving another author of the review. The risk of bias was assessed according to the following domains: (1) Random sequence generation (to determine if the method of randomization sequence generation was adequate, such as random number tables, computer-generated random numbers, minimization, drawing lots, etc.), (2) Allocation concealment (to determine if appropriate methods were used to conceal allocation, such as central randomization and opaque envelopes, sequentially numbered and sealed), (3) Blinding of participants and personnel, (4) Blinding of outcome assessors. Blinding was considered separately for self-reported subjective outcomes (pain, function, treatment success, quality of life) and objective outcomes (such as abstinences, adverse events, disability). For example, for a non-blinded assessment of outcomes, the risk of bias for mortality may differ from that for a pain scale reported by participants), (5) Incomplete outcome data, (6) Selective reporting of outcomes, (7) Other potential threats to validity, such as inadequate analyses in crossover trials, baseline imbalance in important factors, inappropriate or unequal application of co-interventions.

Each potential source was classified as high risk, low risk, or unclear risk regarding bias, taking into account the lack of information or uncertainty about the potential for bias. The numbers generated by the "Risk of Bias Tool" were presented in the "Risk of Bias" table, providing summarized assessments of the risk of bias, along with justifications for each judgment. A summary of the risk of bias across different studies was addressed for each listed domain.

Additionally, information regarding the risk of bias related to unpublished data or correspondence with researchers was recorded in the "Risk of Bias" table. In the context of analyzing the main results, the impact of missing data was taken into account.

When assessing the treatment effects, the risk of bias of the contributing studies for each outcome was taken into account. The numbers generated by the "Bias Risk" tool were presented to summarize the results of the evaluations.

The review was conducted in accordance with the published protocol, and any deviations from this protocol were reported in the "Differences between protocol and review" section of the study. The results from each trial were presented as point estimates, along with the mean and standard deviation (SD) for continuous outcomes and risk ratios (RR) with corresponding 95% confidence intervals (CIs) for dichotomous outcomes.

Dichotomous data, such as RRs or odds ratios from Peto, were analyzed when the outcome was a rare event (approximately less than 10%), and 95% CIs were used.

For continuous data, when different scales were used to measure the same conceptual outcome (e.g., disability), standardized mean differences (SMDs) were calculated, along with corresponding 95% confidence intervals (CIs). The SMD was expressed as an MD on a typical scale (e.g., 0 to 10 for average pain) by multiplying the SMD by a typical SD among people (e.g., the SD of the control group at the baseline of the most representative trial).^7^

In the Comments column of the ‘Summary of Results’ table, the absolute percentage difference, the relative percentage change from baseline, and the number needed to treat for one additional beneficial outcome (NNTB), or the number needed to treat for one additional harmful outcome (NNTH) were reported (only NNTB and NNTH were provided when the outcome showed a statistically significant difference between treatment groups).

For dichotomous outcomes, such as adverse events, NNTB or NNTH was calculated from the event rate of the control group and the relative risk using the Visual Rx NNT calculator.^8^ NNTB was calculated for continuous measures using the Wells calculator, available on RevMan Web. The minimum clinically important difference (MCID) was used in the calculation of NNTB or NNTH; an MCID of 1.5 points on a 10-point scale for pain was assumed, and 10 points on a 100-point scale for function or disability were used as input into the calculator.^9^

For dichotomous outcomes, the absolute risk difference was calculated using the risk difference statistic (in RevMan Web), and the result was expressed as a percentage. For continuous outcomes, the absolute benefit was calculated as the improvement in the intervention group minus the improvement in the control group (MD), in the original units. The results were then presented as percentages.

The relative percentage change was calculated for dichotomous data as Risk Ratio - 1. For continuous outcomes, the relative difference was calculated as the absolute benefit (MD) divided by the mean baseline of the control group.

Additionally, the combined analysis of the studies was conducted using R programming software, version 4.3.1, with the meta package. Six meta-analyses were performed: the first four used only studies with similar treatment methods, while the fifth used all works to estimate the results, and the sixth included studies that presented the average pain scores.

The researchers of the study were contacted by email to verify the main characteristics of the study and obtain missing numerical data from the results whenever possible (e.g., when a study is identified only as an abstract or when data are not available for all participants). Otherwise, considering that missing data introduces serious biases, the impact of including such studies in the overall assessment of results was explored through a sensitivity analysis. For dichotomous outcomes (e.g., number of withdrawals due to adverse events), the withdrawal rate was calculated using the number of participants randomized in the group as the denominator.^7^ For continuous outcomes (e.g., mean change in pain score), the MD or SMD was calculated based on the number of participants analyzed at that time. If the number of participants analyzed was not presented for each time point, the number of participants randomized in each group at baseline was used.^7^

Whenever possible, the missing standard deviations of other statistics, such as standard errors, confidence intervals, or *p-values*, were calculated using the methods recommended in the Cochrane Handbook for Systematic Reviews of Interventions.^7^ When it was not possible to calculate the standard deviations, they were imputed from other studies with meta-analysis. Any assumptions and imputations were described to handle missing data and explore the effect of imputation through sensitivity analyses.

Clinical and methodological diversity was assessed among the participants, interventions, and outcomes of the included studies to determine if the meta-analysis was appropriate, as observed in the data extraction tables. Statistical heterogeneity was assessed by visual inspection of the forest plot to identify clear differences in outcomes between studies, using the statistical tests I² and Chi². The interpretation of an I² value ranges from 0% to 40% as "not important", 30% to 60% as "moderate heterogeneity", 50% to 90% as "substantial heterogeneity", and 75% to 100% as "considerable heterogeneity". As noted in the Cochrane Handbook for Systematic Reviews of Interventions, the importance of I² depends on (i) the magnitude and direction of effects and (ii) the strength of evidence for heterogeneity.

The Chi² test where p ≤ 0.10 indicates evidence of statistical heterogeneity. If substantial heterogeneity is identified, this was reported and possible causes were investigated.^7^

A funnel plot was created and examined to explore possible small-study biases. When interpreting funnel plots, the various possible reasons for the asymmetry of the plots were analyzed and related to the review's results.

To assess outcome reporting bias, the trial protocols were checked against the published reports. For studies published after July 1, 2005, the Clinical Trials Registry on the International Clinical Trials Registry Platform of the World Health Organization was examined.^10^ It was assessed whether there is selective reporting of outcomes. The following questions and main comparisons were considered: Are opioid analgesics more effective than placebo or no treatment? Do anti-inflammatory medications provide additional benefits when combined with other interventions (e.g., analgesics)? Are analgesics more effective than standard therapies such as the use of glucocorticoids, NSAIDs, or others? Is one analgesic more effective than another?

The main outcomes and comparisons of the review were presented in ‘Summary of Results’ tables that provide important information about the quality of evidence, the magnitude of the effect of the examined interventions, and the sum of available data on outcomes: reported by participants as pain relief of 30% or more; mean overall pain; function; participants’ global assessment of treatment success; quality of life; number of participant dropouts due to adverse events; and number of participants who experienced an adverse event, as recommended by Cochrane.^7^ The moments included were: pre and post-surgical approaches, after three weeks to six weeks in the tables, except for withdrawals (failures) and adverse events, which were reported at the end.

Two reviewers independently assessed the quality of the evidence. The five GRADE considerations (study limitations, effect consistency, imprecision, indirectness, and publication bias) were used to assess the quality of a body of evidence regarding studies contributing data for the pre-specified outcomes in meta-analyses, and to report the quality of evidence as high, moderate, low, or very low using GRADEpro GDT software.^11^ All decisions to downgrade the quality of studies were reported using footnotes and comments to help the reader understand the review when necessary.

The following sensitivity analyses were conducted: assessment of the robustness of pain and function results for selection and detection biases, removal of trials in secondary analyses with inadequate or unclear allocation concealment to assess the effect of selection bias, and trials with unclear or inadequate blinding of participants to assess the effect of detection bias. Assessment of the effect of including imputed data and assumption-based data. For the interpretation of the results, we were aware of distinguishing between a lack of evidence of effect and a true lack of effect. The conclusions were based solely on the results of the quantitative or narrative synthesis of the studies included in this review. Recommendations for practice and implications for future research were not addressed, and instead, the remaining uncertainties in the field were outlined.

## RESULTS

**Figure 1:**
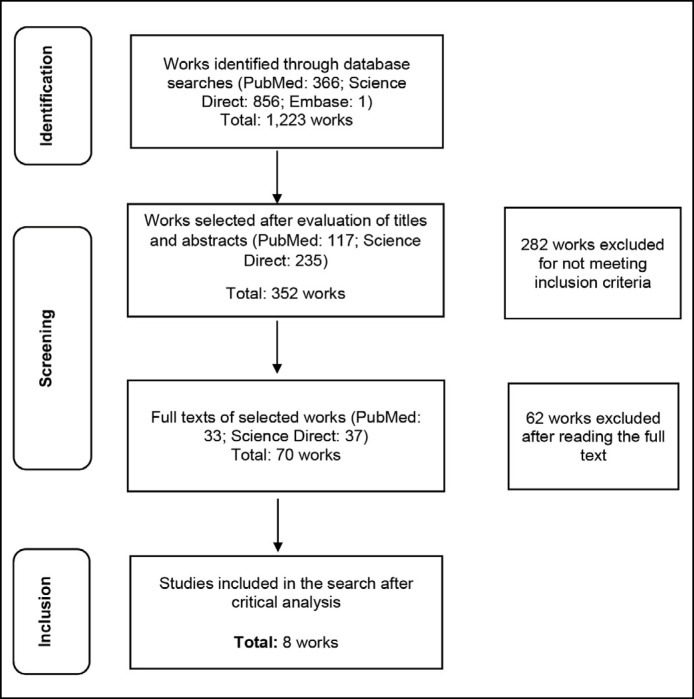
Flowchart illustrating the schematic representation of methods for identifying, screening, assessing eligibility, and including works in the review, adapted from the PRISMA protocol.

Source: Author (2024).

Initially, 1,223 articles were identified in the initial search. The summary of the article selection process is presented in Figure 1. After evaluating the titles and abstracts, followed by the selection and detailed analysis of the articles, 11 studies were deemed eligible to be included in this systematic review.

This systematic review was conducted in accordance with the PRISMA recommendations, as illustrated in Figure 1.

The studies included in this systematic review consisted of randomized clinical trials that investigated the effects of various medications on postoperative pain control after RCR.

The total sample comprised 703 patients. A predominance of male participants was observed in most studies, with an average age range of participants varying from 50.0 to 59.9 years. The methodological characteristics of the selected studies are detailed in Table 3.

**Table 3:** Summary of demographic data and pharmacological intervention of all included studies.

Author/ Year	Study Type	Intervention Groups	Gender (M/F)	Age (years)	Doses
Chillemi et al., 2023.^12^	Randomized Clinical Trial	A: 38 B: 38 C: 38	62/52	59±9	A: Paracetamol 1000mg, 1 tablet as needed, maximum 3 times a day / 7 days B: Paracetamol 500 mg + Codeine 30 mg 1 tablet 3 times a day / 7 days C: Paracetamol 500 mg + Ibuprofen 150 mg 1 tablet 2 times a day / 7 days
Tangtiphaiboontana et al., 2021.^13^	Randomized Clinical Trial	Ibuprofen: 51 Placebo: 50	Ibuprofen: 29/22 Placebo: 29/21	Ibuprofen: 57.7±10.8 Placebo: 56.9±13.8	Ibuprofen: ibuprofen 400 mg every 8 hours / 14 days continuously + opioid Placebo: placebo + opioid
Yang et al., 2022.^14^	Randomized Clinical Trial	Pre-operative analgesia: 53 Post-operative analgesia: 53	Pre-operative analgesia: 31/22 Post-operative analgesia: 26/27	Pre-operative analgesia: 50.0±10.0 Post-operative analgesia: 52.4±9.0	Pre-operative analgesia: 400 mg of Celecoxib 2 hours before surgery, and then 200 mg of Celecoxib at 4 hours, 12 hours, 24 hours, 36 hours, 48 hours, 60 hours, and 72 hours after surgery Post-operative analgesia: 400 mg of Celecoxib 4 hours after surgery, and 200 mg of Celecoxib at 12 hours, 24 hours, 36 hours, 48 hours, 60 hours, and 72 hours after surgery
Singh et al., 2021^15^	Randomized Clinical Trial	G1: 21 G2: 18 G3: 18	G1: 10/11 G2: 03/15 G3: 07/11	G1: 56.67±11.45 G2: 57.11±8.51 G3: 59.72±8.23	G1: 5 mg of Oxycodone every 6 hours, and 1,000 mg of acetaminophen every 6 hours if necessary G2: 5 mg of Oxycodone every 6 hours if necessary G3: 1,000 mg of acetaminophen every 6 hours pre-surgery, and every 8 hours until the 5th post-operative day and 5 mg of oxycodone if necessary
Bang et al., 2010.^16^	Randomized Clinical Trial	Placebo: 23 Gabapentin: 23	Placebo: 18/08 Gabapentin: 14/09	Placebo: 59.5±6.2 Gabapentin: 56.3±8.5	Placebo: placebo identical in appearance to gabapentin Gabapentin: 300 mg of gabapentin 2 hours before surgery
Ahn et al., 2016^6^	Randomized Clinical Trial	Pregabalin: 30 Control: 30	Pregabalin: 13/17 Control: 13/17	Pregabalin: 55±9 Control: 51±12	Pregabalin: 150 mg of pregabalin Control: placebo capsules
Su et al., 2022^17^	Randomized Clinical Trial	Control: 60 Intervention: 60	NR	NR	Control: placebo Intervention: oral duloxetine
Alaia et al., 2022^18^	Randomized Clinical Trial	Control: 50 Experimental: 49	NR	18 to 75 years	Control: placebo Experimental: 25 mg of CBD 3 times a day if <80 kg, or 50 mg of CBD 3 times a day if >80 kg, for 14 days post-operative

Legend: NR: Not reported; M: male; F: female; PO: Post-operative; CBD: cannabidiol. Source: Author (2024).

The included studies encompassed a variety of pain management strategies following RCR, highlighting the diversity of existing approaches in the literature, including the use of analgesics, anti-inflammatory agents, specific medications, and multimodal protocols.

Table 4 highlights the adverse events and effects on pain observed in the studies.

**Table 4:** Summary of adverse effects and pain effects of medications in the post-operative period.

Author/ Year	Adverse Events	Effects on Pain
Chillemi et al., 2023.^12^	No adverse signs/symptoms were highlighted during the administration of the medication.	The use of oral anti-inflammatory is a viable strategy for controlling post-operative pain. The combination of paracetamol with ibuprofen reduces pain, as seen in the VAS, and promotes early recovery of passive range of motion.
Tangtiphaiboontana et al., 2021.^13^	No adverse signs/symptoms were highlighted during the administration of the medication.	The post-operative use of ibuprofen reduces the need for opioids and decreases the patient's pain levels in the first week post-operatively. The use of ibuprofen does not increase the risk of re-rupture.
Yang et al., 2022.^14^	The most commonly occurring adverse events were nausea, constipation, vomiting, drowsiness, and dizziness in both groups.	Pre-operative administration of celecoxib alleviates acute pain and improves perceived satisfaction.
Singh et al., 2021^15^	The commonly reported side effects associated with post-operative medication included nausea, constipation, and drowsiness.	The use of perioperative paracetamol significantly reduced opioid consumption and improved overall pain control.
Bang et al., 2010.^16^	The side effects associated with the medication were nausea, dizziness, and respiratory difficulty with no difference between the groups.	The use of 300 mg of gabapentin could reduce the post-operative VAS score with fewer side effects.
Ahn et al., 2016^6^	The occurrence of complications related to the multimodal analgesic regimen, including sedation, headache, dizziness, and blurred vision, was similar between the groups.	The numerically assessed pain scores were significantly lower in the Pregabalin group at 6, 24, and 48 hours postoperatively.
Su et al., 2022^17^	The incidence of nausea and vomiting in the duloxetine group was significantly higher than in the placebo group.	Duloxetine resulted in a significant reduction in pain in the first 2 postoperative days, but the reduction was not clinically significant.
Alaia et al., 2022^18^	NR	Orally absorbed cannabidiol demonstrated an acceptable safety profile and showed promise in reducing pain in the immediate perioperative period after RCR compared to the control.

Legend: RCR, rotator cuff repair; VAS, visual analog scale for pain; NR, not reported. Source: Author (2024).

Overall, we found that the interventions employed were well-tolerated, with some studies reporting only mild adverse effects, such as nausea, vomiting, dizziness, headache, and other transient symptoms. It is noteworthy that, despite the diversity of approaches, all investigated strategies showed positive results regarding effective postoperative pain control.

This consistency in favorable outcomes highlights the robustness of the available therapeutic options, providing not only analgesic efficacy but also a good safety margin in terms of tolerability. These observations reinforce the feasibility and clinical utility of the reviewed interventions for pain management in the context of rotator cuff repair.

**Figure 2:**
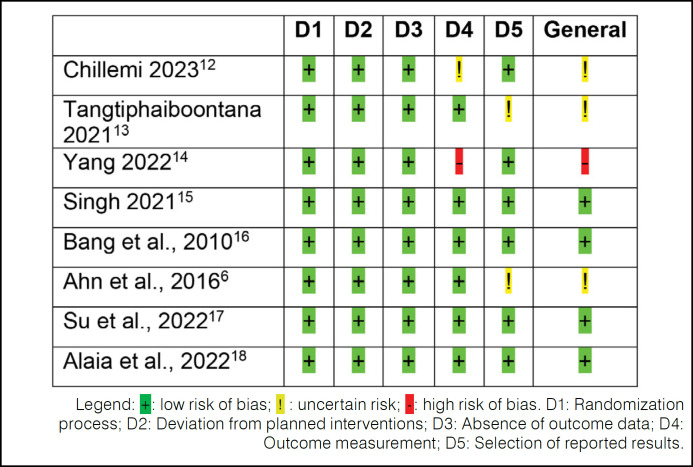
Assessment of methodological risk of bias of the studies according to the Risk-of-bias assessment for randomized trials by Cochrane.^7^

Regarding methodological rigor, 50.0% of the studies presented a low risk of bias, while 50.0% presented a moderate or high risk of bias. (Figure 2)

Among the main factors related to this assessment, we found two studies that presented insufficient or inconsistent data in measuring the outcome (Chillemi et al.^12^ Yang et al.^14^). Two other studies reported incomplete outcomes (Ahn et al,^6^ Tangtiphaiboontana et al.^13^).

**Figure 3:**
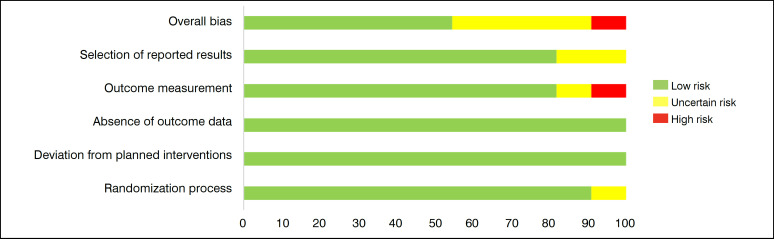
Assessment of risk of bias regarding the analysis categories of all grouped studies.

Some items were rated as "uncertain risk" due to insufficient information that would allow a correct understanding of the methodological process. Figure 3 presents the graphical distribution of potential risks concerning the evaluated items.

**Figure 4:**
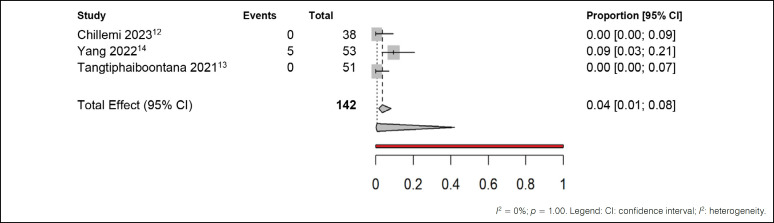
Meta-analysis of adverse effects outcomes in patients treated with NSAIDs in the postoperative period.

In addition, five estimates were made using the same methodology regarding the number of patients who experienced any adverse effect during treatment, regardless of whether the patient presented only one or more symptoms. We employed a random-effects meta-analysis model for binary outcome data, which addresses the objective mentioned at the beginning of the paragraph. The first comparison was made with studies that used NSAIDs in the postoperative period, as shown in Figure 4.

In the analysis conducted, we observed a low level of heterogeneity (I² = 0%) among the included studies (Chillemi et al.^12^ Yang et al.^14^) and (Tangtiphaiboontana et al.^13^), indicating a remarkable consistency in the results obtained. This uniformity was attributed to the clear cause identified: two studies reported the complete absence of adverse effects in the treated group, suggesting a disparity in the pain treatment methods adopted. Despite this discrepancy, when considering the common effect observed in all studies, it was found that only 4% of patients experienced some type of adverse effect. Notably, when adjusting for random effects, this percentage was reduced to 1%. Thus, although the variation in results was clarified by the lack of adverse effects in some studies, the overall analysis revealed a low incidence of those associated with treatment.

**Figure 5:**
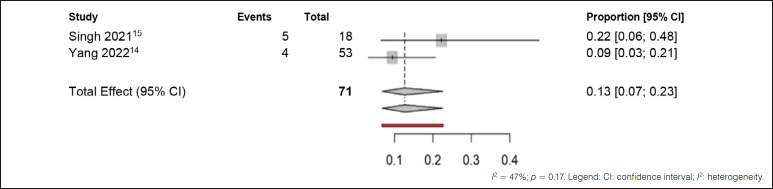
Meta-analysis of adverse effects outcomes in patients treated with medications in the preoperative period.

The second comparison was made with studies that used medications in the preoperative period, as shown in Figure 5.

The statistical analysis revealed moderate heterogeneity among the included studies, with an I² value of 47%, indicating considerable variation in the results. Despite this variation, when considering the common effect observed in all studies (Singh et al.^15^ and Yang et al.^14^), it was found that approximately 13% of patients experienced some type of adverse effect with the medications administered in the preoperative period. This proportion remained unchanged at 13%, even after adjusting for random effects. Therefore, although there was moderate heterogeneity among the studies, the overall incidence of adverse effects remained consistent.

**Figure 6:**
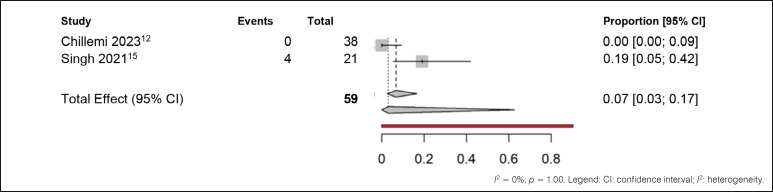
Meta-analysis of adverse effects outcomes in patients treated with analgesics and opioids.

The third comparison was made with studies that treated patients with analgesics and opioids, as shown in Figure 6.

**Figure 7:**
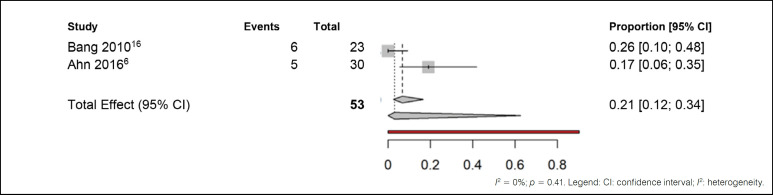
Meta-analysis of adverse effects outcomes in patients treated with anticonvulsants.

In the comparative analysis between the studies of Chillemi et al.^12^ and Singh et al.^15^, a remarkable consistency in results was observed, reflected by a low level of heterogeneity (I² = 0%). This uniformity was attributed to the complete absence of adverse effects in the treated group in the study by Chillemi et al.^12^, possibly due to differences in the pain treatment methods employed, which included the use of analgesics and opioids. Despite this discrepancy, when considering the common effect observed in all studies, it was found that only 7% of patients experienced adverse effects. After adjusting for random effects, this proportion was reduced to 3%. Therefore, despite the variations in results attributed to the lack of adverse effects in the study by Chillemi et al.,^12^ the overall analysis suggests a low incidence of adverse events related to treatment. The fourth comparison was made with studies that treated patients with anticonvulsants. (Figure 7)

In comparing the results of the studies by Bang et al.^16^ and Ahn et al.^6^, which investigated the use of anticonvulsants, low heterogeneity was observed (I² = 0%). Notably, when considering the common effect among these studies, about 21% of patients experienced adverse effects. After random adjustments, this proportion remained stable at 21%.

**Figure 8:**
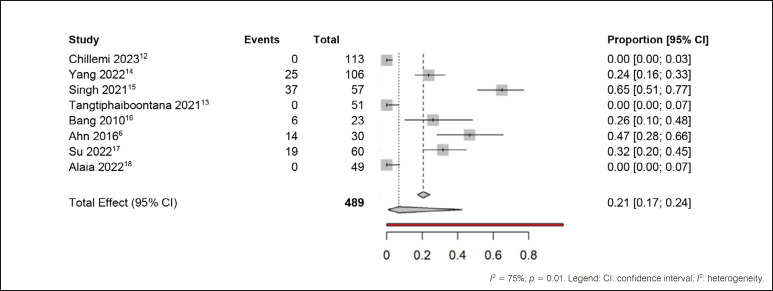
Meta-analysis of adverse effects outcomes in patients.

The fifth analysis used all previous works for an overall meta-analysis of adverse effects, as shown in Figure 8.

When comparing the results of all included studies, significant heterogeneity was evidenced (I² = 75%), with substantial statistical relevance (p < 0.01). Notably, when analyzing the shared effect among these studies, it was found that approximately 21% of patients experienced adverse effects. After random adjustments, this proportion stabilized at 7%, indicating a significant decrease in the incidence of adverse events.

**Figure 9:**
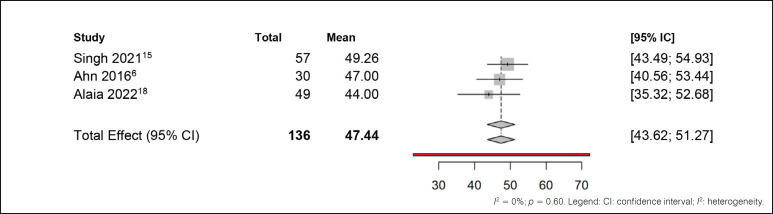
Meta-analysis of adverse effects outcomes in patients.

Finally, the last meta-analysis employed a single means (Meta-analysis of single means) with random effects, as only three of the collected studies used a control group and reported the mean and standard error of the pain score for that group. The selected model enabled the computation of an overall average across all studies that reported the mean and standard error of the pain score, including those that did not use a control group. (Figure 9) The I² statistic indicated low heterogeneity, which was statistically non-significant (p = 0.60), justifying the adoption of the random effects model in the analysis. We observed that the overall mean pain in the studies was 47.44, as demonstrated in Figure 9. he results of the meta-analysis corroborate the effectiveness of oral medications in reducing the mean pain of patients.

## DISCUSSION

The systematic review provides a comprehensive and comparative analysis of the effectiveness of various medications in managing postoperative pain in patients undergoing rotator cuff repair (RCR), which remains a significant challenge for the care team. In this context, effective management of postoperative pain remains a significant challenge in clinical practice, considering the diversity of available strategies.

Several factors contribute to pain in this context, and the review reflects this complexity by incorporating a range of approaches that involve different classes of medications. The examined literature emphasizes the importance of selecting medications carefully to optimize pain control, acknowledging that the superiority of one approach over another has not been conclusively established (Lee et al.^1^).

From this perspective, we agree with Chillemi et al.^12^, who emphasize the use of a combination of paracetamol with codeine or ibuprofen as an effective strategy for controlling pain after RCR, resulting in better reductions on the Visual Analog Scale (VAS) and an earlier recovery of range of motion. This approach emphasizes the importance of selecting suitable medications to enhance patient comfort during the early stages of recovery.

Similarly, Tangtiphaiboontana et al.^13^ explored the postoperative use of non-steroidal anti-inflammatory drugs (NSAIDs), such as ibuprofen, highlighting the significant reduction in the need for opioids and the lower total consumption of morphine equivalents in the first postoperative week. In addition to pain control, the group that received ibuprofen showed short-term functional improvements, with a lower rate of re-rupture indicating safety in its use beyond pain relief.

The perioperative use of paracetamol in multimodal analgesia in patients undergoing RCR was utilized by Singh et al.^15^ The significant reduction in opioid consumption, along with better overall pain control, is highlighted as a crucial role of paracetamol in postoperative management, providing comprehensive and sustainable relief.

In the study by Yang et al.^14^ the authors focus on the preoperative administration of Celecoxib, demonstrating a significant reduction in pain scores in the first 12 hours and on the first day after surgery. This observation suggests that preoperative Celecoxib plays a crucial role in the immediate alleviation of pain, offering relief in the early stages of recovery.

Agreeing with these studies, a systematic review conducted by Toma et al.^15^ highlighted that the analgesic protocol for RCR should encompass an arthroscopic approach, administration of paracetamol, use of NSAIDs, incorporation of dexamethasone, and application of regional analgesic techniques, with opioids considered as rescue analgesics. Furthermore, they emphasized that systemic analgesia should involve the administration of paracetamol and NSAIDs in both the preoperative and intraoperative periods, with continuity in the postoperative period.

Additionally, the efficacy of preoperative gabapentin is highlighted by Bang et al.^16^ with a significant reduction in pain scores at 2, 6, and 12 hours postoperative. Gabapentin can be used as an alternative to cyclooxygenase-2 inhibitors. This result suggests that gabapentin used in the first 24 hours after rotator cuff repair contributes to a more comfortable postoperative experience, reducing pain intensity immediately after RCR.

Ahn et al.^6^ have demonstrated the efficacy of pregabalin as an adjunct in postoperative analgesia following arthroscopic shoulder surgeries. Preoperative administration as an adjunct to patient-controlled analgesia (IV-PCA) resulted in lower fentanyl consumption during the first 48 hours postoperative, highlighting its role in reducing opioid consumption and improving the postoperative experience. In addition to improving postoperative pain, there was no increase in side effects related to the use of pregabalin and a lower need to resort to rescue opioids.

In 2022, Su et al.^17^ analyzed the effects of duloxetine, demonstrating a significant reduction in pain in the first 48 hours postoperative, although its use did not reach clinical significance. This observation highlights the immediate efficacy of duloxetine, although its clinical relevance may be questioned. In contrast, Alaia et al.^18^ investigated orally absorbed CBD, highlighting a significant reduction in pain scores on the first postoperative day, resulting in greater patient satisfaction. This finding suggests that CBD may play a beneficial role in perioperative pain management.

The findings of these studies offer a comprehensive and insightful perspective on pain management strategies following RCR. Furthermore, the choice between preoperative or perioperative medications has been shown to significantly influence immediate outcomes and patient satisfaction, emphasizing the need for a personalized approach.

The common trend of reducing opioid consumption reflects a growing search for safer strategies in postoperative pain management after RCR, aligned with concerns about the adverse effects associated with opioids. However, it is crucial to recognize the need to consider individual patient characteristics when selecting a pain management strategy, ensuring long-term safety and efficacy. Moreover, the importance of additional research is emphasized to validate these findings and provide more specific guidance for clinical practice, contributing to significant advances in the field of postoperative pain treatment after RCR.

## CONCLUSION

This systematic review and meta-analysis showed that the postoperative use of ibuprofen reduces the need for opioids and decreases patient pain levels in the first week postoperative, and its use did not increase the risk of a new rupture. Specific medications, such as Celecoxib, Gabapentin, Duloxetine, and Pregabalin, have demonstrated efficacy in reducing opioid consumption without compromising functional outcomes. Duloxetine provided immediate pain relief, and orally absorbed cannabidiol proved to be promising. There is no definitively established oral protocol in the medical literature, and the choice of drug depends exclusively on the surgeon's preference. There is still a lack of evidence for a more robust routine recommendation of interventions related to perioperative analgesia in the surgical treatment of rotator cuff injuries.
